# [Corrigendum] miR‑199b‑5p serves as a tumor suppressor in renal cell carcinoma

**DOI:** 10.3892/etm.2024.12422

**Published:** 2024-02-09

**Authors:** Yulin Lai, Jing Quan, Canbin Lin, Hang Li, Jia Hu, Peijie Chen, Jinling Xu, Xin Guan, Weijie Xu, Yongqing Lai, Liangchao Ni

Exp Ther Med 16:436–444, 2018; DOI: 10.3892/etm.2018.6151

Following the publication of the above article and a corrigendum (10.3892/etm.2018.6333) that was concerned with changes in the authorship and affiliation details on the paper, an interested reader has drawn to the authors’ attention that, in Fig. 5A on p. 441, the ‘ACHN/cell invasion/miR-199b-5p inhibitor’ and ‘ACHN/cell invasion/NCin’ data panels appeared to show an overlapping section of data, such that they were derived from the same original source when they were intended to show the results from differently performed experiments.

The authors have re-examined their data, and realize that the ‘ACHN/cell invasion/NCin’ data panel was inadvertently selected incorrectly. The corrected version of Fig. 5, now containing the correct data for the ‘ACHN/cell invasion/NCin’ experiment, is shown on the next page. Note that the error made during the compilation of this figure did not affect the overall conclusions reported in the paper. All the authors agree with the publication of this corrigendum, and are grateful to the Editor of *Experimental and Therapeutic Medicine* for offering them the opportunity to publish this. They also apologize to the readership for any inconvenience caused.

## Figures and Tables

**Figure 5:**
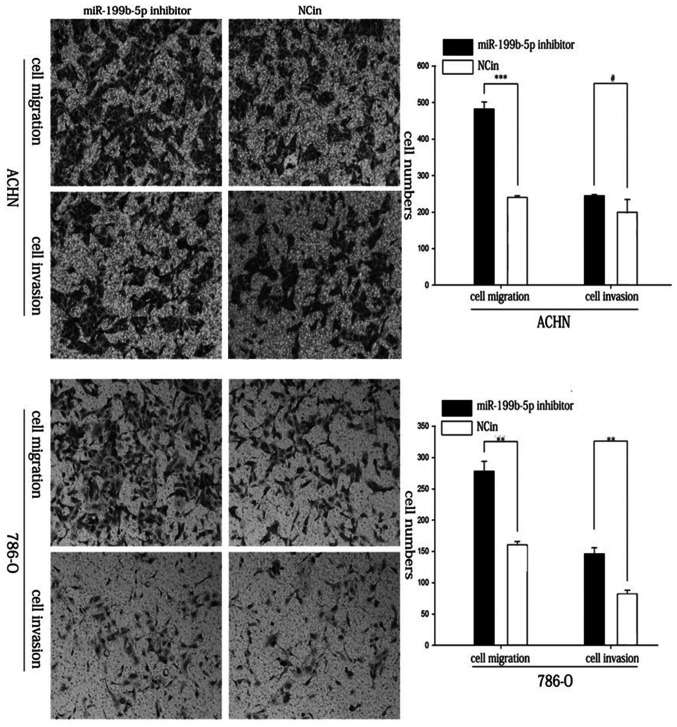
Transwell assay of ACHN and 786-O cells. (A) Representative images of the lower side of the Transwell membranes with invaded and migrated ACHN cells. (B) The numbers of migratory and invasive ACHN cells were quantified. (C) Representative images of the lower side of the Transwell membranes with invaded and migrated 786-O cells (magnification, ×100). (D) The numbers of migratory and invasive 786-O cells were quantified. ^**^P<0.01, ^***^P<0.001. miR, microRNA; NCin, negative control inhibitor.

